# The Teaching of Stoicism

**Published:** 1908-08-01

**Authors:** 


### The Hospital

A Journal of

The Medical Sciences and Hospital Administration.

New Series. No. 73, Vol. III. [No. 1145, Vol. XLIV.] SATURDAY, AUGUST 1, 1908.

### The Teaching of Stoicism.

Although philosophers and moralists in all ages have dilated at large upon the advantages to be derived from mental, as opposed to intellectual, schooling, there is little evidence that the world in general sets itself to profit by their wise sayings. Indeed it has become a common maxim of scoffers and cynics that those who in their writings are most sublime and equal-minded are least able to abide with fortitude the trifling discomfitures of everyday life, such as the breaking of a bootlace or the loss of half a sovereign. Armchair philosophy of this unworthy sort has gone far to bring discredit upon a faculty which is not only vital to a happy existence in the case of certain temperaments, but is capable of cultivation to an extent little realised, especially by those who are most in need of it. We have on numerous occasions in these columns deplored the neglect of mental influences in relation to disease which makes so conspicuous a gap in the schedule of medical education in our times. It is perfectly true to say that to this neglect in particular we have to attribute the victories of charlatans and the amazing growth of pseudo-medical religious cults like "Christian Science." For it is seldom that a man turns to irregular medicine until he has exploited in vain the more recognised channels of relief from suffering, and it is idle folly for us to feel affronted because a patient does not accept as final our inability to cure him. It would become us far better to analyse the influences which others have used with success when our own methods have failed; to attempt to systematise them and thus make them readily available for future service.

Now when one comes to contemplate the kind of person which supplies Christian Science with its victories one finds a type which is exceedingly common and in many respects admirable. It is no doubt a neurotic type, sympathetic, emotional, conscientious. These people (they are mostly, but not by any means always, women) are distinguished by characteristics in the main kindly, but exaggerated and uncontrolled. They speak in superlatives, and think in superlatives. It is no wonder then that they feel, so to speak, in superlatives also. Moreover, people thus temperamentally endowed are particularly prone to the multitudinous vagaries of the physical system which we are wont to embrace under the term "functional disturbances": unless indeed it be that these are common to us all but are not represented in the consciousness of more phlegmatic individuals. Whichever explanation be adopted, the fact remains that these unfortunates, to whom every twinge is an agony and every least anxiety a terror, have unending opportunities for the exercise of their exhausting reaction to trifling stimuli, both physical and mental, and live in a world of suffering to which the distresses of mere physical disease are a bagatelle. Bright and vivacious in company, when alone they are often plunged in gloomy contemplation of their vague unhappiness, and intensify it by their brooding. Commonly they defer for a long period any attempt at seeking medical advice, chiefly because they do not know what to complain of; but at last, under the influence of some localisation of pain, they visit a doctor. He, if a thorough-going materialist, will treat the symptom, wash out the stomach, sew up the kidneys, or rectify the position of the uterus, as the case may be. If more imaginative, he will see through the disguise of local pain and do his best to reassure the patient; will recognise her as neurotic, and dose her with bromides. In either case the inevitable happens, and after a period of temporary improvement there follows a relapse, perhaps to a condition worse than ever under the influence of disappointment. After one or two such experiences enters the *deus ex machina* in the shape of a friend who under similar circumstances has been cured by Christian Science. The invalid, whose sole immediate object is restoration to health, and who has perhaps proved her anxiety in this direction by submitting herself in the past to various operative procedures, embraces the new hope held out to her, listens with attention to the exposition of the religious treatment, and, if persuaded, enters a new world of health and happiness to the astonishment of her circle and her own abiding thankfulness.

This picture, which we honestly think is not overdrawn, is no rarity. What, then, has happened to this patient whom regular medicine has so egregiously failed to cure? Clearly the cure is psychical, the outcome of a change in her mental attitude towards pain under the influence of religious consolation. Whereas previously her consciousness was perpetually on the watch for sensory impressions, and this, too, in an attitude of most anxious expectation which gave them their fullest value when they came, her advance in mental placidity now enables her to disregard them. She has, in fact, grown stoical in response to a religious dogma, and has compassed peace of mind. This, without disrespect, we take to be the psychical explanation of the indisputable cures of Christian Science. Those who practise this variety of "healing" incur the well-deserved censure of thoughtful people in so far as their irrationalism and ignorance of medicine robs them of all discrimination in the choice of subjects for treatment. They thus bring odium and ridicule upon themselves and danger upon their clients, as is testified from time to time in the public Press. None the less we may learn from them, if we will, how much may be done by mental therapeutics. It must not be thought that such a change of mental attitude as has been described above postulates a basis of religious enthusiasm. If this were so we might despair of its limitations and leave the business to those whose religious convictions are unassailable. But simple stoicism permits of inculcation by the direct road of reason, creed and dogma quite apart. To be persuaded of this it is only necessary to read the "Morals" of Epicurus—a much-abused man but a healthy stoic nevertheless, though the formal Stoics would have none of him—or the "Meditations" of Marcus Aurelius Antoninus. Indeed, an eclectic perusal of these works might with advantage be prescribed to many patients who are at this moment taking bromides in large quantities.

